# Geographical variability and factors associated with caesarean section delivery in India: a comparative assessment of Bihar and Tamil Nadu

**DOI:** 10.1186/s12889-021-11750-4

**Published:** 2021-09-21

**Authors:** Avijit Roy, Pintu Paul, Pradip Chouhan, Margubur Rahaman, Nanigopal Kapasia

**Affiliations:** 1 Department of Geography, University of Gour Banga, Malda, West Bengal 732103 India; 2 Department of Geography, Malda College, Malda, West Bengal 732101 India; 3 Centre for the Study of Regional Development, School of Social Sciences, Jawaharlal Nehru University (JNU), New Delhi, 110067 India; 4 International Institute for Population Sciences (IIPS), Govandi Station Road, Deonar, Mumbai, 400088 India

**Keywords:** Caesarean delivery, Caesarean section, Socio-demographic factors, Pregnancy, Public-private sector, India, NFHS-4

## Abstract

**Background:**

Caesarean section delivery is a major life-saving obstetric surgical intervention for mothers and babies from pregnancy and childbirth related complications. This paper attempts to investigate the geographical variations and correlating factors of caesarean section delivery in India, particularly focusing on the states of Bihar and Tamil Nadu, accounting for one of the lowest and highest prevalence states of caesarean section delivery respectively.

**Methods:**

This study is based on secondary data, collected from the fourth round of the National Family Health Survey (NFHS-4), 2015–16. We utilized 190,898 women aged 15–49 years who had a living child during the past 5 years preceding the survey. In this study, caesarean section delivery was the outcome variable. A variety of demographic, socio-economic, and pregnancy- and delivery-related variables were considered as explanatory variables. Descriptive statistics, bivariate percentage distribution, Pearson’s Chi-square test, and multivariate binary logistic regression models were employed to draw the inferences from data.

**Results:**

Of participants, about 19% of women had undergone caesarean section delivery in the country**.** The state-wise distribution shows that Telangana (60%) followed by Andhra Pradesh (42%) and Tamil Nadu (36%) represented the topmost states in caesarean delivery, while Bihar (7%), Madhya Pradesh (10%), and Jharkhand (11%) placed at the bottom end. Multivariate logistic models show that the likelihood of caesarean delivery was higher among older women (35–49 years), women with higher levels of education, Muslims, women belonging to the upper quintiles of the household wealth, and those who received antenatal care (ANC), experienced pregnancy loss and delivery complications. Moreover, the odds of caesarean section delivery were remarkably greater for the private health sector than the public health sector in both focused states: Bihar (odds ratio [OR] = 12.84; 95% confidence interval [CI]: 10.90, 15.13) and Tamil Nadu (OR = 2.90; 95% CI: 2.54, 3.31).

**Conclusion:**

Findings of this study suggest that improvement in female education, providing economic incentives, and spreading awareness through mass media could raise the caesarean section delivery among women whose vaginal delivery could be unsafe for them as well as for their babies. Moreover, providing adequate ANC and well-equipped public healthcare services would facilitate caesarean delivery among needy women.

## Background

Caesarean section delivery is a major life-saving obstetric surgical intervention for mothers and babies from pregnancy and childbirth related complications [1, 2]. This vital clinical procedure is performed to avoid obstetric complications and thereby averts the incidence of maternal and neonatal deaths. A number of obstetric complications such as dystocia, foetal distress, breach births, post-term pregnancy, multiple pregnancy, and pregnancy-induced hypertension are recognized as reasonable motives behind caesarean section deliveries [2].

Approximately 18.5 million caesarean births have been recorded each year globally, accounting for 19.1% of total births, which is beyond the cut-off recommended by the World Health Organization (WHO) [3, 4]. Among high-income countries, the caesarean delivery rate is generally higher: 30.3% in the United States, 30% in Australia, 28% in Germany, 26% in Canada, and 22% in the United Kingdom [5]. On the other hand, the caesarean delivery rate is relatively less in low-income countries: 23% in Bangladesh [6], 5% in Nepal [3], 20% in Pakistan [7], and 11.4% in Ghana [8]. However, the increasing rate of caesarean section deliveries has become a matter of serious concern among public health policymakers and practitioners during the recent period and India is not an exception [8]. In India, the rate of caesarean section delivery is dramatically increased from 3% in 1992–93 to 17% in 2015–16. In terms of regional variations, south Indian states have recorded substantially higher levels of caesarean deliveries in comparison to north India [9, 10].

Studies have indicated that various socio-demographic, economic, cultural, and psychological factors influence caesarean section deliveries [10, 11]. Among socio-economic factors, place of residence, education, *caste* (hierarchical social groups), and economic status of the household are some of the important determinants of caesarean delivery [6, 11]. Concerning cultural factors, the health-seeking behaviour of women during pregnancy plays a key role in shaping caesarean section delivery [7, 12]. In addition, various psychological causes such as fear related to prolonged labour and vaginal delivery pain reinforce women’s preferences for caesarean delivery [7]. Studies have also documented the importance of reproductive care including pregnancy- and delivery-related factors in caesarean section delivery. A study conducted in Pakistan found that antenatal care (ANC) of mothers is positively correlated with caesarean delivery [7]. Moreover, pregnancy complication of women increases the likelihood of undergoing caesarean section delivery [7]. In India, an emerging body of research has provided clear and strong evidence about the rising rate of caesarean section deliveries in private sector health facilities, where the gap between the public health sector and the private health sector in caesarean section delivery is continuously amplifying [13–15].

India has made remarkable achievements in institutional delivery over recent decades. The Indian government has initiated several *Safe Motherhood* programmes including *Janani Suraksha Yojana* (JSY) to improve the coverage of institutional delivery in the country, particularly among vulnerable sections by providing economic incentives. Despite several maternal healthcare initiatives, the incidence of maternal mortality for some of the states is still alarming. For instance, maternal mortality rate is considerably higher in low-performing states such as– Uttar Pradesh (197 deaths per 100,000 live births) and Bihar (149 deaths per 100,000 live births) than the national average (113 deaths per 100,000 live births) and south Indian states (67 deaths per 100,000 live births) [16]. A study on maternal mortality in India observed the highest maternal mortality rate in rural areas of poorer states (397 per one million live births), in which 82% of maternal deaths have been attributed to direct obstetric causes [17]. It is noteworthy to mention that maternal mortality could be averted by caesarean section delivery and thus women who have obstetric complications should undergo caesarean delivery.

Despite several studies that have investigated the factors contributing to caesarean section delivery, systematic evidence is still lacking towards understanding the determining factors and formulating effective policies to address the increasing rate and uneven geographical distribution of caesarean section deliveries in India. With this backdrop, this study attempts to examine the geographical variations and socio-demographic characteristics and pregnancy- and delivery-related factors associated with caesarean section delivery among Indian women using the most recent nationally representative survey. Further, our study focuses on two socio-culturally distinct states, namely Bihar and Tamil Nadu to understand the differential influence of various factors on caesarean section delivery.

## Methods

### Data source

We used data from the fourth round of the National Family Health Survey (NFHS-4), conducted in 2015–2016. It is a large-scale nationally representative sample survey covering all 29 states and seven union territories of India. The survey is conducted by the International Institute for Population Sciences (IIPS) under the stewardship of the Ministry of Health and Family Welfare (MoHFW), Government of India. The survey provides essential information on population, health, and family welfare. A two-stage stratified sampling design was adopted for the selection of the participants. In total, 28,586 clusters (primary sampling units) were chosen, of which fieldwork was completed for 28,522 clusters. The 2011 Census enumeration served as the sampling frame for the selection of clusters. In the first stage, clusters have been chosen using probability proportional to cluster size. In the second stage, 22 households were selected from each cluster with an equal opportunity systematic selection from the household listing. A detailed description of the sampling design and survey procedure has been provided in the national report of the NFHS-4 [18].

### Study participants

The NFHS-4 interviewed 699,686 eligible women aged 15–49 years with a response rate of 97% in 601,509 households. For the country-level analysis, the participants were limited to women aged 15–49 years who had given birth to at least one child during the last 5 years preceding the survey (*n* = 190,898). Furthermore, we utilized a subset of samples for the analysis of selected states: Bihar (*n* = 16,822) and Tamil Nadu (*n* = 6181).

### Outcome variable

Caesarean section delivery was the outcome variable in this study. In the NFHS-4 survey, women were asked whether *the baby delivered by caesarean section*, *that is, did they cut your belly open to take the baby out?*” Women who responded ‘*yes*’ considered as “*delivered by caesarean section*” (coded as ‘1’); otherwise considered as ‘*vaginal delivery*’ (coded as ‘0’).

### Explanatory variables

A range of demographic, socio-economic, and pregnancy- and delivery-related variables were included as explanatory variables. Demographic characteristics include women’s age (15–24, 25–34, and 35–49 years), age at marriage (< 18, and ≥ 18 years), and birth order (first, second, third, and fourth or more). The socio-economic variables included in this study are the place of residence (rural and urban), women’s education (no education, primary, secondary and higher), religion (Hindu, Muslim, and other), *caste* (*Scheduled Caste, Scheduled Tribe, Other Backward Class*, and others [*forward caste*]), wealth quintile (poorest, poorer, middle, richer, and richest), and exposure to mass media (no, partial, and full exposure).

It is noteworthy to mention that the wealth index is a measure of a household’s standard of living. The wealth index was computed by the ownership of household assets, housing characteristics, and accessibility to basic services using principal component analysis (PCA). The survey generated scores for each individual and divided them into five quintiles, ranging from the poorest (1) to the richest (5). Women’s exposure to mass media was measured from the frequency of listening to the radio, reading newspapers/magazines, and watching television.

In addition, we included variables related to maternity care including pregnancy health: number of ANC visits, place of delivery, pregnancy loss, and delivery complications. ANC of mothers is a vital component of maternity care. ANC visits of the women were classified as no visit, 1–3 visits, and four or more visits. The place of delivery was categorized as public and private health facilities. Public facilities include government/municipality hospitals, government dispensaries, urban health clinics/urban health posts (UHP)/urban family welfare centers (UFWC), community health centers (CHC)/rural hospitals/block primary health centers (BPHC), PHC/additional PHC, sub-centers and other public sector health facilities, while private facilities include hospital/maternity home/clinics, other private sector health facilities, and NGOs or trust hospital/clinics. Pregnancy loss was dichotomized as “yes” (women who experienced abortion, miscarriage, or stillbirth) and “no” (women who did not experience any pregnancy loss). Similarly, delivery complications were grouped as a binary response: “yes” (women who suffered from prolonged labor, breech presentation, or excessive bleeding) and “no” (women who did not suffer from any complications during delivery).

### Statistical analysis

Descriptive statistics were presented to understand the distribution of study participants for India and the selected states: Bihar and Tamil Nadu. We presented geographical patterns in the prevalence of caesarean delivery by geographical regions and states. Bivariate analysis was conducted to examine the nature of the association between caesarean delivery (outcome variable) and selected explanatory variables. The sample weight was applied to estimate the percentages. Furthermore, Pearson’s chi-square was performed to test the level of significance in the association between outcome and explanatory variables. Finally, multivariate logistic regression models were applied to determine the factors associated with caesarean section delivery for India and selected states separately. The significant explanatory variables in the bivariate analysis were included in the multivariate analysis. The selection of variables in the multivariate logistic regression model was set at *p* < 0.05. We checked for multicollinearity between explanatory variables using variance inflation factor (VIF) prior to performing the multivariate analysis and found no evidence of multicollinearity problem. The regression results are presented by odds ratio (OR) and a 95% confidence interval (CI). All Statistical analyses were performed using STATA version 14.0 (StataCorp LP, College Station, TX, USA).

## Results

### Descriptive statistics

Table 1 presents the distribution of the study participants. On average, about one-third of the respondents were in the younger age group (aged 15–24 years). Over half of the women (56%) got married before they reach 18 years in Bihar, while one in every five women (20%) was child-married in Tamil Nadu. The proportionate share of study participants decreased with birth order. In India, about three-quarters of the respondents (75%) lived in rural areas. In Bihar, over half of the women (55%) were illiterate, while the corresponding figure was only 6% in Tamil Nadu. The majority of the participants were Hindu and two-fifth belonged to the *Other Backward Class* category. The distribution of the study population also differed across the wealth quintiles, ranging from 25% in the poorest to 15% in the richest quintile. About one in four sample women had no exposure to mass media in the country. Over half of women (57%) had no mass media exposure in Bihar, while it is only 2% in the case of Tamil Nadu. Nearly half of the participants (47%) received four or more ANC visits in the country with a considerable difference between Bihar and Tamil Nadu. The majority of the respondents (72%) had their delivery in the public sector with a 7% gap between Bihar and Tamil Nadu. In India, about 16 and 11% of the respondents ever experienced pregnancy loss and delivery complications, respectively.

**Table 1:** Descriptive statistics of the study participants, NFHS-4 (2015–16)

	India		Bihar		Tamil Nadu	
Variables	N	%	N	%	N	%
**Women’s age**						
15–24 years	62,082	32.5	5707	33.9	1996	32.3
25–34 years	107,500	56.3	8999	53.5	3829	62.0
35–49 years	21,316	11.2	2116	12.6	356	5.8
**Age at marriage**						
< 18 years	69,751	37.3	9302	55.6	1213	19.8
≥ 18 years	117,078	62.7	7434	44.4	4922	80.2
**Birth order**						
1	61,807	32.4	3878	23.1	2497	40.4
2	62,484	32.7	4442	26.4	2823	45.7
3	33,064	17.3	3644	21.7	710	11.5
4+	33,543	17.6	4858	28.9	151	2.4
**Place of residence**						
Urban	47,833	25.1	1780	10.6	2687	43.5
Rural	143,065	74.9	15,042	89.4	3494	56.5
**Women’s education**						
Illiterate	55,165	28.9	9223	54.8	343	5.6
Primary	26,712	14.0	2036	12.1	430	7.0
Secondary	88,871	46.6	4764	28.3	3978	64.4
Higher	20,150	10.6	799	4.8	1430	23.1
**Religion**						
Hindu	138,343	72.5	14,038	83.5	5534	89.5
Muslim	29,309	15.4	2767	16.5	323	5.2
Others	23,246	12.2	17	0.1	324	5.2
**Caste**						
Scheduled Caste	35,170	19.3	3747	22.5	1849	30.0
Scheduled Tribe	37,889	20.8	543	3.3	111	1.8
Other Backward Class	74,060	40.7	9980	59.8	4113	66.8
Others	34,705	19.1	2410	14.5	88	1.4
**Wealth quintile**						
Poorest	46,782	24.5	9191	54.6	201	3.3
Poorer	43,739	22.9	4004	23.8	933	15.1
Middle	38,393	20.1	2028	12.1	1914	31.0
Richer	33,212	17.4	1215	7.2	2042	33.0
Richest	28,772	15.1	384	2.3	1091	17.7
**Exposure to mass media**						
No exposure	49,374	25.9	9597	57.1	114	1.8
Partial exposure	126,910	66.5	6195	36.8	5022	81.3
Full exposure	14,614	7.7	1030	6.1	1045	16.9
**Number of ANC visit**						
No visit	33,642	17.8	7011	41.8	509	8.3
1–3	65,964	34.9	7299	43.5	621	10.2
4+	89,438	47.3	2458	14.7	4991	81.5
**Place of delivery**						
Public sector	105,615	71.7	8474	74.2	4069	66.6
Private sector	41,643	28.3	2946	25.8	2044	33.4
**Pregnancy loss**						
No	161,055	84.4	14,572	86.6	5306	85.8
Yes	29,843	15.6	2250	13.4	875	14.2
**Delivery complications**						
No	165,076	88.6	8135	48.4	1128	18.3
Yes	21,325	11.4	8687	51.6	5053	81.8

### Geographical variations in caesarean section delivery

**Fig. 1:**
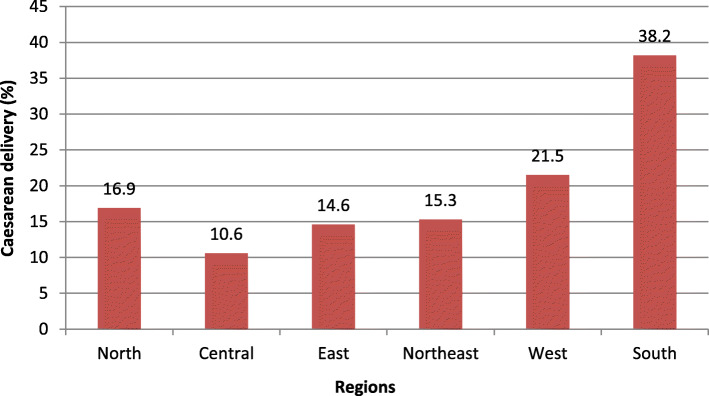
Percentage distribution of caesarean section delivery by geographical regions, NFHS-4 (2015–16)

Nearly one-fifth of sample women (19%) had undergone caesarean section delivery in India. The southern region exhibited the highest prevalence of caesarean delivery (38%), followed by the western region (22%). On the other spectrum, the central region (11%) followed by the east region (15%) had the lowest prevalence of caesarean section delivery. It is also apparent that the patterns of caesarean delivery largely correspond to the level of socio-economic development of the country (Fig. 1).

**Fig. 2:**
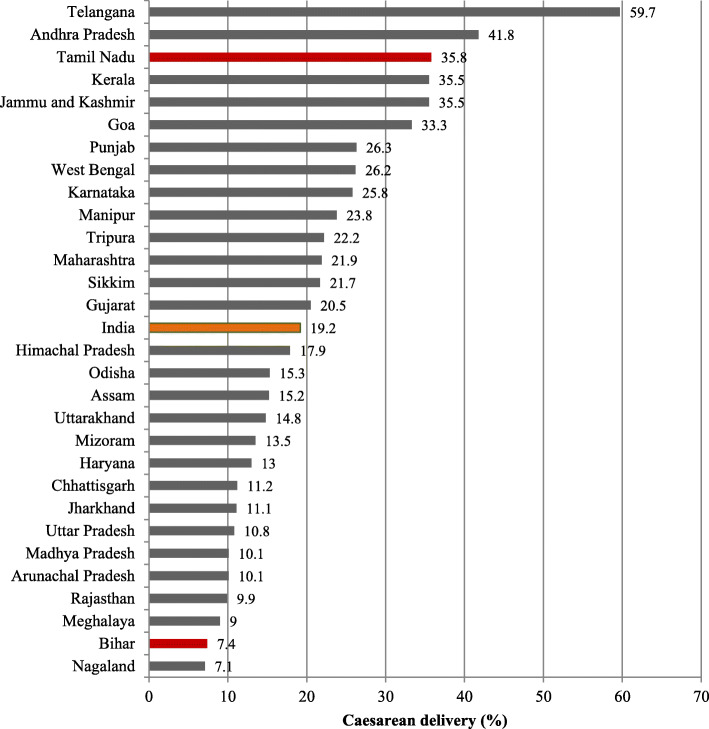
Prevalence of caesarean section delivery across Indian states, NFHS-4 (2015–16)

In terms of state-wise distribution, we found a considerable variation in caesarean deliveries, ranging from 7.1% in Nagaland to 59.7% in Telangana. The focused states in this study, namely Tamil Nadu (35.8%) and Bihar (7.4%), represent one of the topmost and bottommost prevalence states, respectively (Fig. 2).

### Prevalence of caesarean section delivery by explanatory variables

Table 2 shows the prevalence of caesarean section delivery by selected explanatory variables. The percentage of caesarean delivery was lower among the older women (aged 35–49 years) as compared to their younger peers (aged 15–24 years and 25–34 years) in India. Likewise, the rate of caesarean delivery was low among older women in Bihar, while it had an increasing trend with the increasing age of women in Tamil Nadu. Concerning age at marriage, the prevalence of caesarean delivery was considerably higher among those women who married at 18 years or older as compared to those who married below 18 years. A similar relationship between age at marriage and caesarean delivery was observed for both selected states. Birth order had a negative association with caesarean section delivery where the prevalence of caesarean delivery was found to be decreased with the increasing birth order.

**Table 2:** Prevalence of caesarean section delivery by selected explanatory variables, NFHS-4 (2015–16)

	India		Bihar		Tamil Nadu	
Variables	%	***p***-value	%	***p***-value	%	***p***-value
**Women’s age**		< 0.001		< 0.001		< 0.001
15–24 years	18.3		8.5		31.4	
25–34 years	20.2		7.5		38.5	
35–49 years	16.8		4.3		44.4	
**Age at marriage**		< 0.001		< 0.001		< 0.001
< 18 years	12.1		5.6		27.7	
≥ 18 years	24.1		9.7		37.8	
**Birth order**		< 0.001		< 0.001		< 0.001
1	27.4		14.1		39.2	
2	21.9		8.8		36.8	
3	11.0		5.1		23.4	
4+	4.2		2.4		14.9	
**Place of residence**		< 0.001		< 0.001		0.001
Urban	30.4		16.3		37.5	
Rural	14.5		6.4		34.2	
**Women’s education**		< 0.001		< 0.001		< 0.001
Illiterate	6.9		4.2		26.8	
Primary	12.6		5.6		31.3	
Secondary	23.1		11.2		34.5	
Higher	39.8		28.2		42.5	
**Religion**		< 0.001		0.146		< 0.001
Hindu	19.4		7.6		35.0	
Muslim	16.9		6.4		39.3	
Other	23.8		1.6		46.0	
**Caste**		< 0.001		< 0.001		< 0.001
Scheduled Caste	16.4		4.9		31.8	
Scheduled Tribe	9.5		2.9		30.8	
Other Backward Class	19.2		7.0		37.4	
Others	26.0		14.3		46.4	
**Wealth quintile**		< 0.001		< 0.001		< 0.001
Poorest	5.2		4.0		35.3	
Poorer	10.9		7.0		27.2	
Middle	20.6		11.2		33.5	
Richer	28.7		22.0		37.5	
Richest	37.2		31.9		42.1	
**Mass media exposure**						
No exposure	5.8	< 0.001	4.4	< 0.001	32.9	0.483
Partial exposure	22.8		11.0		36.2	
Full exposure	30.6		14.1		34.1	
**Number of ANC visit**		< 0.001		< 0.001		0.055
No visit	6.4		3.6		33.2	
1–3	12.1		6.9		31.7	
4+	27.7		20.6		36.7	
**Place of delivery**		< 0.001		< 0.001		< 0.001
Public sector	13.2		2.9		27.6	
Private sector	43.2		34.1		52.8	
**Pregnancy loss**		< 0.001		0.01		< 0.001
No	18.7		7.3		34.7	
Yes	22.2		8.6		42.6	
**Delivery complications**		< 0.001		< 0.001		< 0.001
No	17.9		5.4		58.3	
Yes	22.4		9.2		30.7	

Note: *P*-values are derived from Pearson’s chi-square test between the outcome and explanatory variables

Participants who lived in rural areas were less frequent to deliver in caesarean section as compared to those living in urban areas. A positive association between women’s educational attainment and caesarean delivery was found for both states. However, the prevalence gap in caesarean delivery between the respondents with no education and higher education was more pronounced in the case of Bihar than in Tamil Nadu. Overall, the percentage of caesarean delivery was slightly higher among Hindus as compared to Muslim women. Caste-wise distribution demonstrates that caesarean delivery rate was found to be highest among *forward caste* women, followed by *Other Backward Class* category. The prevalence of caesarean delivery was significantly differed across wealth groups, ranging from 5% among the poorest quintile to 37% among the richest quintile at the national level. Moreover, we found a large variation in the prevalence of caesarean delivery by the wealth quintile in Bihar (ranging from 4% in the poorest to 32% in the richest quintile). Women’s exposure to mass media had a positive association with caesarean delivery. In Bihar, a significantly higher percentage of participants who had partial or full access to mass media reported undergoing caesarean section delivery as compared to those who had no access to mass media.

There was a positive relationship between the number of ANC visits and caesarean delivery, in which caesarean delivery was higher among mothers who received four or more ANC visits as compared to those who received less than four ANC visits. Concerning public-private distribution, the prevalence of caesarean delivery was remarkably higher in the private health sector than in the public sector at the national level (43% vs. 13%). In Bihar, the percentage of caesarean section delivery was 34% in the private sector, while it was only 3% in the public sector. In Tamil Nadu, over half (53%) of the deliveries occurred in the private sector under caesarean section. A significantly higher percentage of women who experienced pregnancy loss had undergone caesarean section delivery as compared to those who did not experience any pregnancy loss. Similarly, mothers who had faced delivery complications at the time of childbirth were more likely to experience caesarean section delivery in Bihar, while an inverse relationship was found between delivery complications and caesarean delivery in Tamil Nadu.

### Factors associated with caesarean section delivery

Table 3 presents the results of the multivariate logistic regression models assessing the factors associated with caesarean section delivery for India and two selected states– Bihar and Tamil Nadu.

**Table 3:** Multivariate logistic regression models assessing the factors associated with caesarean section delivery in India, Bihar, and Tamil Nadu, NFHS-4 (2015–16)

	India		Bihar		Tamil Nadu	
Variables	OR	95% CI	OR	95% CI	OR	95% CI
**Women’s age**						
15–24 years (ref.)						
25–34 years	1.40**	1.34–1.45	1.30**	1.09–1.55	1.49**	1.30–1.72
35–49 years	2.25**	2.10–2.40	1.47*	1.05–2.04	2.02**	1.52–2.67
**Age at marriage**						
< 18 years	0.92**	0.88–0.96	0.96	0.82–1.12	0.87	0.74–1.03
≥ 18 years (ref.)						
**Birth order**						
1 (ref.)						
2	0.74**	0.72–0.77	0.69**	0.58–0.83	0.89	0.78–1.01
3	0.44**	0.41–0.46	0.49**	0.39–0.61	0.43**	0.34–0.53
4+	0.26**	0.24–0.28	0.31**	0.23–0.41	0.23**	0.14–0.38
**Place of residence**						
Urban (ref.)						
Rural	0.85**	0.82–0.88	0.77*	0.62–0.95	1.03	0.91–1.17
**Women’s education**						
Illiterate (ref.)						
Primary	1.12**	1.05–1.20	0.88	0.68–1.13	0.95	0.67–1.34
Secondary	1.26**	1.20–1.33	0.81*	0.66–0.99	0.99	0.75–1.30
Higher	1.31**	1.22–1.40	0.85	0.63–1.14	0.96	0.71–1.31
**Religion**						
Hindu (ref.)						
Muslim	1.12**	1.07–1.18	1.03	0.84–1.27	1.08	0.83–1.42
Other	1.03	0.97–1.08	1.1	0.09–13.45	1.26	0.98–1.62
**Caste**						
Scheduled Caste (ref.)						
Scheduled Tribe	0.77**	0.73–0.82	0.64	0.35–1.17	0.95	0.59–1.50
Other Backward Class	0.89**	0.85–0.93	0.99	0.81–1.21	0.98	0.86–1.13
Others	1.00	0.95–1.05	1.27*	1.00–1.63	1.08	0.66–1.13
**Wealth quintile**						
Poorest (ref.)						
Poorer	1.18**	1.10–1.26	1	0.82–1.22	0.64*	0.45–0.92
Middle	1.53**	1.43–1.63	0.84	0.65–1.07	0.70*	0.50–1.00
Richer	1.61**	1.50–1.73	0.95	0.72–1.25	0.62**	0.43–0.88
Richest	1.45**	1.35–1.57	0.86	0.59–1.24	0.58**	0.40–0.85
**Exposure to mass media**						
No exposure (ref.)						
Partial exposure	1.26**	1.19–1.33	1.17	0.97–1.40	0.97	0.62–1.53
Full exposure	1.48**	1.38–1.59	1.15	0.87–1.52	0.93	0.58–1.50
**ANC visit**						
No visit (ref.)						
1–3	1.14**	1.07–1.22	1.1	0.92–1.33	0.92	0.70–1.20
4+	1.71**	1.61–1.82	1.88**	1.53–2.30	1.06	0.85–1.31
**Place of delivery**						
Public sector (ref.)						
Private sector	3.88**	3.76–4.01	12.84**	10.90–15.13	2.90**	2.54–3.31
**Pregnancy loss**						
No (ref.)						
Yes	1.18**	1.13–1.23	1.03	0.85–1.25	1.29**	1.10–1.52
**Delivery complications**						
No (ref.)						
Yes	1.34**	1.28–1.40	2.17**	1.70–2.77	0.41**	0.37–0.46
Constant	0.06**	0.05–0.06	0.04**	0.03–0.06	0.86	0.46–1.59
LR chi^2^		20,260.3		1947.8		775.3
Prob> chi^2^		0.00		0.00		0.00
Pseudo R^2^		0.1577		0.2644		0.1009
Log-likelihood		−54,093.2		− 2709		− 3455.7

Notes: *OR* Odds ratio, *CI* Confidence interval, *ref.* Reference category

**p < 0.01; *p < 0.05

#### Country-level association

The results show that the likelihood of caesarean delivery increased with the age of women where women aged 25–34 years (OR = 1.40; 95% CI: 1.34, 1.45) and 35–49 years (OR = 2.25; 95% CI: 2.10, 2.40) were significantly more likely to deliver by caesarean section as compared to women aged 15–24 years. Women who married before 18 years were less likely to experience caesarean section delivery (OR = 0.92; 95% CI: 0.88, 0.96) as compared to those who married at 18 years or later. Birth order was negatively associated with caesarean delivery, indicating the odds of caesarean section delivery decreased with birth order. Women with two (OR = 0.74; 95% CI: 0.72, 0.77), three (OR = 0.44; 95% CI: 0.41, 0.46), and four or more birth order (OR = 0.26; 95% CI: 0.24, 0.28) had a lower likelihood of caesarean delivery as compared to those who had first-order birth.

Women living in rural areas were significantly less likely than urban women to undergo caesarean section delivery (OR = 0.85; 95% CI: 0.82, 0.88). The educational attainment of women was found to be positively associated with caesarean section delivery. Women with primary (OR = 1.12; 95% CI: 1.05, 1.20), secondary (OR = 1.26; 95% CI: 1.20, 1.33), and higher (OR = 1.31; 95% CI: 1.22, 1.40) levels of education were more likely to have caesarean delivery as compared to the uneducated women. Religion-wise, Muslim women were 12% more likely to have caesarean section delivery (OR = 1.12; 95% CI: 1.07, 1.18) as compared to Hindus. Women belonged to *Scheduled Tribe* (OR = 0.77; 95% CI: 0.73, 0.82) and *Other Backward Class* (OR = 0.89; 95% CI: 0.85, 0.93) had lower odds of caesarean delivery as compared to *Scheduled Caste* women. The wealth quintile of the household also had a strong positive correlation with caesarean delivery. Compared to the poorest women, the likelihood of caesarean delivery was 1.5 times (OR = 1.45; 95% CI: 1.35, 1.57) higher among the women from the richest quintile. Women’s exposure to mass also had a positive relationship with caesarean delivery, indicating that women who had full exposure to mass media were more likely to undergo caesarean section delivery (OR = 1.48; 95% CI: 1.38, 1.59) than those who had no exposure to mass media.

ANC visits of women acted as an enabling factor of caesarean section delivery in which women who received four or more ANC visits were 1.7 times (OR = 1.71; 95% CI: 1.61, 1.82) more likely to have caesarean section delivery as compared to those who did not receive ANC service. Women who had delivered in the private health institution were associated with almost four-fold increased odds of caesarean section delivery (OR = 3.88; 95% CI: 3.76, 4.01) than those who delivered in the public sector. Women who had experienced pregnancy loss (OR = 1.18; 95% CI: 1.13, 1.23) and delivery complications (OR = 1.34; 95% CI: 1.28, 1.40) were more likely to undergo caesarean delivery than their counterparts.

#### Bihar and Tamil Nadu

Similar to India results, women aged 25–34 years and 35–49 years were more likely than women aged 15–24 years to undergo caesarean section delivery in both states (Bihar and Tamil Nadu); however, the association appeared to be much stronger in Tamil Nadu. Birth order had a negative relationship with caesarean delivery in both states. In Bihar, women living in rural areas were less likely than urban areas to have a caesarean birth (OR = 0.77; 95% CI: 0.62, 0.95). We found a very weak relationship between women’s educational attainment and caesarean delivery in Bihar, and no significant relationship was found in the case of Tamil Nadu. Religious affiliation of participants had no significant association with caesarean section delivery in both states. In Bihar, women who belonged to *forward caste* were significantly more likely than *Scheduled Caste* to deliver by caesarean section (OR = 1.27; 95% CI: 1.00, 1.63). Unlike India results, the wealth status of the household had a negative relationship with caesarean delivery of women in Tamil Nadu. However, the association between the wealth quintile and caesarean delivery was found to be insignificant in Bihar. We found no significant relationship between women’s exposure to mass media and caesarean delivery in both states.

In Bihar, ANC of mothers was positively related to caesarean delivery, in which women who received four or more ANC visits were almost twice more likely to undergo caesarean section delivery (OR = 1.88; 95% CI: 1.53, 2.30) as compared to women who did not visit for ANC service. Place of delivery was found to be a strong correlate of caesarean delivery. In Bihar, women who delivered in the private sector were associated with almost 13-fold more odds of caesarean delivery (OR = 12.84; 95% CI: 10.90, 15.13) as compared to the public sector. In Tamil Nadu, the likelihood of caesarean delivery in the private sector increased by nearly three-fold (OR = 2.90; 95% CI: 2.54, 3.31). In Tamil Nadu, women who had a pregnancy loss were associated with an increased likelihood of having caesarean birth (OR = 1.29; 95% CI: 1.10, 1.52) as compared to women who had no pregnancy loss. Likewise, women having delivery complications were related to higher odds of caesarean delivery (OR = 2.17; 95% CI: 1.70, 2.77) in Bihar, whereas delivery complications had a negative association with caesarean delivery (OR = 0.41; 95% CI: 0.37, 0.46) in Tamil Nadu.

## Discussion

The present study highlights the regional and state-level variations in caesarean section delivery and its associated factors in India and two selected states namely, Bihar and Tamil Nadu. We found noticeable differences in patterns and the likelihood of caesarean section delivery by selected socio-economic, demographic, and maternal health-related explanatory factors in India and selected states. In the present study, state-level patterns suggest that the prevalence of caesarean delivery was almost two times higher in Tamil Nadu (35.8%) and three times lower in Bihar (7.4%) than the nation's average (19.2%). The rate of caesarean section delivery for the national average and Tamil Nadu already crossed the upper limit of WHO-recommended population-based caesarean section delivery threshold (5–15%) for developing countries [19]. A previous study also found a similar finding [20]. The prevalence of caesarean delivery in south Indian states like Tamil Nadu and Kerala was considerably higher due to the high demand for caesarean section delivery [20, 21]. All possible caesarean section demand-side factors are substantially higher in Tamil Nadu as well as other southern states as found in many previous studies [20, 22]. First, reproductive health factors associated with caesarean section delivery such as the prevalence of obesity, pregnancy complications, and pregnancy termination are significantly higher in Tamil Nadu than in Bihar. Second, factors related to demographics such as late marriage and childbirth at later ages may also increase the caesarean section deliveries in southern states like Tamil Nadu. It is evidenced that south Indian states are characterized by high levels of female literacy, lower fertility, and higher age at marriage [23]. Third, many economic, socio-cultural, and development-related factors such as modernization and high levels of urbanization, wealthier economic backgrounds, and higher status of women could lead to a higher probability of undergoing caesarean section delivery in Tamil Nadu. Fourth, factors related to health care services such as accessibility to maternity healthcare utilization (ANC, safe delivery, PNC) in both private and public facilities are substantially better in Tamil Nadu as compared to eastern states like Bihar. In the southern region, the availability of well-equipped healthcare facilities in both public and private health sectors increased the likelihood of caesarean section delivery [20]. Similarly, Kathuria and Raj TP (2020) also suggested that the transition of high caesarean section delivery in southern states is mainly due to the increasing trend of institutional delivery in these states [24]. Besides all these socio-economic, demographic, and maternal healthcare factors, the socio-cultural diffusion of caesarean section delivery enhances the positive attitudes toward availing caesarean section delivery in Tamil Nadu and other southern states as suggested by previous studies [22, 24, 25]. Therefore, the significant role of all these associated factors drives the chances of caesarean section delivery among women in Tamil Nadu. However, the high rate of caesarean section delivery is a matter of serious concern for Tamil Nadu and other top-performing states [19, 26]. In addition, caesarean section delivery is significantly associated with high out-of-pocket expenditure, catastrophic health expenditure, and the burden of healthcare facilities. If the upward trend of caesarean delivery continues, it will be alarming especially for socio-economically disadvantaged communities in top-performing caesarean section delivery states [26]. In Bihar, poor socioeconomic status, deprivation in accessing healthcare facilities, the persistence of early marriage practice, and low maternity healthcare utilization decreased the probability of availing caesarean section delivery [22, 27].

Findings of the present study show that women’s age, age at first marriage, birth order, place of residence, *caste*, educational attainment, wealth status, and all selected reproductive health-related explanatory variables (ANC visit, pregnancy loss, place of delivery, and pregnancy complication) are significant predictors of caesarean section delivery in India. The results are similar to many previous studies conducted in India and elsewhere [28]. In the present study, maternity care and pregnancy health-related factors principally explained the utilization of caesarean section delivery more than socio-cultural factors in Bihar [19]. The comparative patterns of caesarean section delivery by selected explanatory variables between Tamil Nadu and Bihar showed that the gaps in caesarean section delivery by rural-urban residents, illiterate-higher education, and poor-rich economic groups were substantially higher in Bihar than in Tamil Nadu. The findings of this study are consistent with previous researches conducted in India [20, 22].

With regard to demographic factors, our findings show that the likelihood of caesarean section delivery was higher among older women (35–49 years) than younger ones. Modugu et al. (2012) suggested that the high prevalence of multiple births, obesity, and pregnancy complications among older women increase the probability of undergoing caesarean section delivery [29]. The higher prevalence of caesarean delivery was also found among women who married at 18 years or later as compared to their child-married peers (married before 18 years). The chances of maternity complications are significantly higher among women who married at later ages or had first childbirth at later ages found in previous studies [30, 31]. Therefore, the possible reason for the high prevalence of caesarean section delivery among older women may be due to maternity complications. Moreover, women who married in their adulthood may have a higher decision-making autonomy in terms of health-seeking behaviour than those who married at their adolescent age. Mothers in their first birth are more likely to prefer caesarean section delivery probably due to avoid complications of vaginal delivery and labour pain. On the other hand, mothers who had experienced vaginal delivery at their first birth might reject caesarean delivery for the next children due to overcoming fear related to childbirth.

We found that *caste* differentials in caesarean section delivery are prominent in Bihar. In Bihar, the prevalence of caesarean section delivery among the women who belonged to the *upper caste* was four times higher than the *scheduled caste/tribe*, indicating the existence of caste inequality. A previous study also found notable *caste* differences in institutional deliveries in Bihar [32]. The main reasons for low coverage of caesarean deliveries in rural Bihar may be due to high illiteracy, poor socio-economic conditions, lack of awareness, and substantial hinders to utilize maternity care services [20, 33]. Srivastava et al. (2020) found that the high clusters of early marriage and adolescent childbirth are spatially correlated with the low prevalence of caesarean section delivery in Bihar [20]. Apart from socio-economic obstacles, the poor infrastructure for maternal health care services is another major challenge for meeting the demand of caesarean section delivery and institutional delivery in Bihar, especially in rural areas. The likelihood of caesarean delivery was lower among women who belonged to *Scheduled Tribe* which concurs with previous studies conducted in India [27, 34]. Moreover, delivery at home is common among tribal women owing to lack of information and inaccessibility, leading to high maternal and child deaths. Due to the impoverishment and extreme levels of marginalization, tribal communities do not have access to adequate healthcare facilities. A study conducted in Madhya Pradesh reported that about two-fifth of all maternal deaths have occurred among tribal women, which are directly linked to childbirth by unskilled professionals [35].

Women’s education was not found to be a strong correlating factor of caesarean section delivery in Tamil Nadu and Bihar. In India, we observe elevated odds of caesarean section delivery among women with higher levels of education. This result is similar to a previous study [22]. Women with higher levels of education may have better decision-making autonomy to access obstetric care and have an opportunity to manage the risk of childbirth-related complications [8]. However, the relationship between women’s educational status and caesarean section delivery varies with the study population and geographical boundary. For example, the correlation between education and caesarean section delivery was found positive in Bangladesh [36, 37], Brazil [38], Pakistan [7], and Nepal [3], and negative in Iran [39]. Several previous studies from Bangladesh [6], Pakistan [7], China [40], Mozambique [41], and Ghana [28], demonstrated a significant association between household wealth status and caesarean delivery. In the present study, we also found that wealth status was positively correlated with caesarean delivery in India. Women from wealthier families may have no financial constraints to undergo caesarean section delivery. However, an inverse relationship was observed between household wealth quintile and caesarean section delivery in Tamil Nadu. In Brazil, a similar finding is reported in which women belonging to the upper wealth quintiles are less likely than lower quintiles to deliver in a caesarean section [42]. Studies have indicated that women’s exposure to mass media had a positive influence on family planning and maternal health care utilization [43–45]. The present study also found that exposure to mass media is one of the facilitators of caesarean delivery. Findings advocate that women who had partial or full media exposure were more likely to undergo caesarean delivery in India.

In our study, ANC visits of mothers had a positive impact on undergoing caesarean section delivery where women who received ANC during pregnancy were more likely to deliver in the caesarean section which is in accordance with other studies [8, 10, 22]. It seems that mothers get motivated to undergo safe delivery during their ANC visits by healthcare staff and that could lead to a positive influence on caesarean delivery. Place of delivery is found to be strongly associated with caesarean section delivery. In India, women delivered in the private health sector were almost four times more likely to have caesarean births compared to the public sector. Similar findings have been reported in other studies [11, 46, 47]. Although caesarean delivery in the private sector was higher in both the states as compared to the public sector, the likelihood of caesarean section delivery in the private health sector was more than four-fold higher in Bihar than in Tamil Nadu. The higher rate of caesarean delivery in the private sector could be due to sufficient modern medical instruments, specialized treatment, adequate medical staff and caretakers, the demand of couples [48, 49]. It is also possible that some of the public health institutions (e.g., government dispensaries) are not well-equipped to perform the caesarean delivery, particularly in socio-economically backward states like Bihar. An earlier study reported that the quality of caesarean section delivery or institutional delivery services in the public health sector is extremely poor in Bihar [50]. Moreover, women having pregnancy complications could increase ANC visits and they are more likely to prefer caesarean section as a safe delivery option [51]. Likewise, women who had ever experienced pregnancy loss were more likely to have a caesarean birth as compared to those who had never experienced pregnancy complications. They prefer caesarean delivery to avoid any complications during childbirth; especially those mothers who had their conception at older ages might drive them quickly to opt for a caesarean section [15, 52].

### Policy implications

The findings of our present study suggest some policy implications. Firstly, southern states crossed the WHO-recommended caesarean delivery threshold point (5–15%). Therefore, there is a need to examine the increasing trend of caesarean section delivery in these states through in-depth investigation. It is imperative to understand whether caesarean section deliveries have been performing for profit maximization in private health care facilities. Secondly, improvements in public healthcare facilities are needed to avoid catastrophic health expenditure among socio-economically deprived sections. Thirdly, striking socio-economic differentials in caesarean section delivery were found in the present study, indicating persistent socioeconomic inequalities in health care services. The government should focus on socio-economically vulnerable sections by providing adequate maternal and child health care services. The socio-economically backward states have very low coverage of institutional delivery as well as caesarean section delivery due to the unavailability of well-equipped health care facilities in the public health care sector. There is an urgent need to avail proper health care facilities by strengthening existing policies to reduce the public-private gap in caesarean delivery. Finally, there is a need for national awareness programmes related to caesarean section delivery which will be helpful to spread appropriate knowledge about the need of undergoing caesarean section delivery among reproductive women.

### Strength and limitations

The present study is based on a nationally representative sample survey of NFHS-4. Therefore, the results of this study could be generalized to the whole country. Additionally, our study focuses on two selected states, namely Bihar and Tamil Nadu, which account for one of the lowest and highest prevalence states respectively in India. Therefore, the findings of this study could be helpful for policymakers and health practitioners to design effective policies and programs to address the vulnerable sections.

The findings of this study should be understood in light of some limitations. First, the collected information is self-reported; therefore, the data are prone to recall bias. Second, due to the cross-sectional nature of data, causal relationships could not be established between the outcome variable and explanatory variables. Third, caesarean delivery can be influenced by many cultural, physiological, and behavioural factors; however, we could not include these factors in the analysis due to the unavailability of information in the dataset. Finally, the data does not provide details of medical reasons for caesarean delivery. Further in-depth qualitative research is needed to understand the driving forces behind caesarean section delivery.

## Conclusion

This study has identified several important factors associated with caesarean section delivery for the whole country as well as two focused states: Bihar and Tamil Nadu. The findings indicate that women having higher levels of education, being a Muslim, belonging to the upper quintiles of wealth, having exposure to mass media, and those who received ANC during the pregnancy period were more likely to deliver in caesarean section. It is also evident that the likelihood of caesarean delivery was substantially higher in the private sector as compared to the public health institutions. Moreover, women who experienced pregnancy loss and delivery complications were more likely to undergo caesarean section delivery.

Our findings suggest that improvement in girls’ education, providing economic incentives to poor families, and spreading awareness through mass media could raise the caesarean section delivery among women whose vaginal delivery could be unsafe for them as well as for their babies. Concerning supply-side factors, providing adequate ANC and well-equipped public healthcare facilities would increase caesarean delivery among needy women. Finally, improved targeting of socio-economically vulnerable women in safe delivery services would reduce their adverse reproductive health outcomes including maternal mortality.

## Data Availability

The dataset analysed during the current study are available in the Demographic and Health Surveys (DHS) repository, https://dhsprogram.com/data/available-datasets.cfm.

## Abbreviations

ANC: Antenatal care
CI: Confidence interval
JSY: Janani Suraksha Yojana
NFHS: National Family Health Survey
PCA: Principal component analysis
PNC: Postnatal care
OR: Odds ratio
SDG: Sustainable Development Goal
